# Association of socio-demographic characteristics, comorbidities, lifestyle habits, and saliva parameters with dental caries in adults with obesity

**DOI:** 10.2340/aos.v84.42485

**Published:** 2025-01-06

**Authors:** Virginie Chuy, Marie Mayoute, Maud Monsaingeon-Henry, Blandine Gatta-Cherifi, Élise Arrivé

**Affiliations:** a CHU de Bordeaux, Pôle de Médecine et Chirurgie Bucco-Dentaire, F-33000 Bordeaux, France; b Univ. Bordeaux, INSERM, BPH, U1219, F-33000 Bordeaux, France; c CHU de Bordeaux, Service Endocrinologie, Diabétologie, Nutrition, Hôpital Leveque, F-33000 Bordeaux, France; d Univ. Bordeaux, Oral Health Department, F-33000 Bordeaux, France; e Univ. Bordeaux, INSERM, Physiopathologie de la balance énergétique et obésité, U1215, F-33000 Bordeaux, France

**Keywords:** Obesity, saliva, dental caries, oral health, MCP regression

## Abstract

**Objective:**

To describe the oral health status of patients with obesity and to explore the socio-demographic characteristics, comorbidities, lifestyle habits (tobacco, alcohol, sweet/acidic diet), and saliva parameters most associated with the dental caries experience.

**Material and methods:**

We conducted a cross-sectional analysis of 204 patients’ data with obesity attending a therapeutic education programme. Caries experience (number of decayed, missing, and filled teeth [DMFT]), periodontal status, oral hygiene, occlusal tooth wear, masticatory inefficiency, and saliva parameters were recorded.

**Results:**

Mean DMFT was 12 ± 7 and was independently associated with age (mean 47 ± 14 years; for 1 standard deviation (SD) increase: β = 0.22, 95% confidence interval [CI] = 0.16–0.29), female sex (72%; β = 3.76, 95% CI = 1.65–5.87), brushing <2 times/day (35%; β = 2.86, 95% CI = 0.81–4.90), salivary buffering capacity (low [46%] vs. normal [40%]: β = 2.02, 95% CI = 0.08–3.95; very low [10%] vs. normal: β = 3.34, 95% CI = 0.31–6.37), and salivary consistency (bubbly [30%] vs. clear [57%]: β = 2.45, 95% CI = 0.52–4.38).

**Conclusions:**

Improving patients’ competencies for oral care, such as teeth brushing twice daily, is necessary to limit the burden of dental caries in individuals with obesity. Acting on salivary composition may also be of interest, and further studies are required to explore the underlying mechanisms.

## Introduction

In 2021, the World Health Assembly adopted a resolution for better oral health, recognising that oral diseases are linked to other noncommunicable diseases, which include cardiovascular disease, diabetes, and obesity [1]. Indeed, people living with obesity usually have a poor oral health status, which includes periodontitis [2] and tooth decay [3], causing diminished quality of life, pain, infections, masticatory discomfort [4], and interactions with the comorbidities of obesity [5, 6].

Tooth decay results from demineralisation caused by acidophilic activity of the oral microbiome, mainly driven by poor hygiene habits and a carbohydrate-rich diet [7]. The shared dietary risk factors between obesity and tooth decay partly explains the propensity of people with obesity to have decayed teeth. However, the spectrum of damage can vary greatly among individuals with obesity, raising the question of other underlying mechanisms. A few studies have reported several common comorbidities of obesity that negatively affect teeth health, including type 2 diabetes and obstructive sleep apnea. Indeed, levels of glucose in saliva and gingival crevicular fluid can increase in patients with diabetes [8], whereas sleep apnea leads to gastroesophageal reflux disorder (GERD), oral dryness, and tooth wear [9], which can directly damage the tooth surface or create a favourable environment for acidophilic microorganisms to thrive, facilitating the development of carious lesions. Some studies have also suggested that obesity induces changes in the saliva composition. Hyposalivation has indeed been associated with body mass index (BMI) [10–15] and having a salivary pH that is too acidic or too basic [10, 16]. These changes could subsequently promote the onset of tooth decay [14, 17].

A large number of obesity-related factors could therefore be associated with dental caries. However, these relations remain debated, and determining which factors are most relevant in this association may help identify at-risk patients and prevent the development of tooth decay in this population. Additionally, although people with obesity represent 17% of adults in France [18], no study has investigated their burden of oral disease or habits relative to preventive practices, and little action has been taken to specifically address their oral health needs.

The objectives of this study were therefore to describe the oral health status and related practices of patients living with obesity and to explore socio-demographic characteristics, obesity-related comorbidities, lifestyle habits, and saliva parameters most associated with the dental caries experience in a hypothesis-generating approach.

## Materials and methods

### Study design and sample

We conducted a cross-sectional analysis of clinical data collected between September 2014 and June 2015 during a systematic oral screening proposed to patients hospitalised in the Endocrinology Unit of the University Hospital of Bordeaux (France) for a 1-week therapeutic education programme. Patients who have access to this programme are receiving care management for obesity and have a BMI ≥ 30 kg/m². Participants were informed that the data collected during the oral screening could be used for research and publication purposes, and provided oral consent.

Analyses included patients with complete data on the caries experience and complete or missing completely at random data on covariates.

In view of the documents at its disposal, the Research Ethics Committee of Bordeaux issued a favourable opinion for the publication of this research (Reference CERBDX–2021–48).

### Oral health status and related practices

The oral health assessment included a questionnaire about the use of dental services, dental pain and oral hygiene practices, and a complete clinical evaluation of the oral cavity conducted by a senior-year dental student (MM) according to the World Health Organization (WHO) recommendations for oral health epidemiological surveys [19]. She performed a standardised examination of the oral soft tissue and teeth using a mouth mirror, explorer, and gauze, and an overhead light while the participant was seated in an armchair in a dedicated room of the endocrinology unit.

Oral hygiene was assessed based on the presence, visible to the naked eye, of debris and calculus, and the mucosa was examined for the presence of lesions. Periodontal status was assessed by the number of non-physiological mobile teeth and the presence of gingival recession (i.e. retraction of the gingival margin below the amelocemental junction) and gingival bleeding (spontaneously or on probing).

For each tooth, root and crown were recorded as sound, decayed (when an unmistakable cavity, undermined enamel, or a detectably softened tissue was present on the root or crown), filled (when ≥1 restoration were present with no caries anywhere on the tooth), missing (when extracted presumably because of caries), or replaced by a removable or a fixed denture. Caries experience was assessed as the number of decayed, missing, and filled teeth (DMFT), computed on 28 teeth, excluding third molars. The Root Caries Index (range, 0–100%) was also computed by dividing the number of decayed and filled roots by the total number of roots exposed in the oral cavity [20].

A masticatory coefficient was calculated by summing values assigned to each natural or prosthetic tooth present in the oral cavity and in occlusion with its antagonist (upper and lower lateral incisors and lower central incisors: 1%, upper central incisors and upper third molars: 2%, premolars and lower third molars: 3%, canines: 4%, first and second molars: 5%). Mastication was considered inefficient when the masticatory coefficient was <30% [21]. Occlusal tooth wear was screened using the Basic Erosive Wear Examination index [22]. The sum of the highest score recorded in each sextant (from 0 [no tooth wear] to 3 [hard tissue loss ≥50% of the surface]) was used to indicate the level of occlusal tooth wear (none [0–2]; low [3–8]; medium [9–13]; and high [14–18]).

Finally, saliva characteristics were assessed using five parameters: resting flow rate (by timing the lower lip labial secretion of saliva), consistency (by a visual inspection of the saliva), stimulated flow rate (by collecting the saliva for 5 min after chewing paraffin gum for 30 s), and pH and buffering capacity by using a salivary test (Saliva-Check Buffer^®^, GC Co., Tokyo, Japan) in contact with the saliva before and after stimulation, respectively. Participants were asked not to drink, eat, or smoke in the preceding hour.

### Socio-demographic and medical characteristics and consumptions

Electronic medical records were systematically completed to ensure medical follow-up during hospitalisation; they yielded data on patient BMI, socio-demographic characteristics (sex, age, no salary or monthly allowance), smoking status, alcohol consumption, and comorbidities (dyspnoea, sleep apnea, diabetes, glycated haemoglobin, GERD, and emesis).

Participants also completed a brief in-house food frequency questionnaire, designed to estimate their consumption of sweet or acidic foods and beverages. Participants were asked to report their frequency of consumption per day or week of 10 categories of food and 7 categories of beverages for what they considered to be their usual consumption period (Supplementary **Methods S1**). They were classified as having a sweet diet if they reported consumption of energy drinks, soft drinks, sweets, ice creams, or fast foods at least once a day; or juice several times a day; or more than two high-carbohydrate foods (spread, jam, sugar, honey, dairy products with sugar, or fruit compotes) each day. Participants were classified as having an acidic diet if they reported consumption of energy drinks, soft drinks, or wine at least once a day, or vinaigrette or citrus fruits more than once a day.

### Statistical analyses

Socio-demographic characteristics, comorbidities, lifestyle habits, and oral health status and related practices were described. We used the mean and SD to represent continuous variables and frequencies and percentages to represent categorical variables.

To investigate any potential selection bias, patients not included in the analyses were compared to those included in terms of BMI, sex, age, and absence of salary or monthly allowance using a chi-square test or a Student’s t-test.

To explore the most relevant characteristics associated with the DMFT among socio-demographic factors, obesity-related comorbidities, lifestyle habits, and saliva parameters, a minimax concave penalty (MCP) for linear regression was performed [23] (see Supplementary Methods S2 for a comprehensive description). The MCP is a penalised method similar to LASSO (Least Absolute Shrinkage and Selection Operator) that performs a parsimonious selection of explanatory variables that are most relevant to the outcome without compromising the model prediction error. Before MCP regression: (1) we identified dependency between explanatory variables: alcohol consumption (strongly associated with smoking status) and salivary pH (strongly associated with other saliva parameters) were excluded. (2) Bootstrap samples were generated to check the stability of variable selection and thus improve precision in the identification of patient characteristics related to the DMFT. (3) Data that were missing completely at random due to the absence of report in the medical records (i.e. age: 3%, salary or monthly allowance: 3%, smoking status: 4%, and diabetes: 1%) or the loss of the collected data (diet: 5% and salivary parameters: 4%) were imputed among bootstraps using multiple imputation by chained equations [24]. Next, the percentages of selection across bootstraps following MCP regression were graphically represented and variables before the first sharp decline were considered as the most relevant to DMFT according to the elbow criterion. To improve our understanding of these findings, exploratory post hoc unpenalised linear regression with these variables as explanatory factors were performed on the imputed original dataset.

All statistical analyses were performed with R software (version 4.0.4), and statistical significance was set at *p* < 0.05.

## Results

Among 316 eligible patients, 216 agreed to participate in the oral clinical screening. Analyses excluded 5 patients for whom computation of the DMFT was not possible due to missing data on tooth status and 7 patients with unexplained missing data for the dietary survey, which could be due to their unwillingness to disclose the content of their diet, and thus could be considered as ‘not at random’ missing values. Patients included in the analyses had more frequently dyspnoea or sleep apnea, diabetes and GERD than those not included (Supplementary Table S1). No differences were observed with regard to BMI, sex, age, or absence of salary or monthly allowance.

Among the 204 patients included in analyses, 72% were female; the mean BMI was 43 ± 7 kg/m² (range, 31–79) and the mean age was 47 ± 14 years (19–75 years). As shown in Table 1, 31% of the participants had no salary or monthly allowance, 39% reported a sweet or acidic diet, and 21% were current smokers; only a few participants were alcohol consumers. With regard to obesity-related comorbidities, most participants had dyspnoea and sleep apnea, 20% had diabetes (including one-third with uncontrolled diabetes, that is, glycated haemoglobin >7%), and 25% had GERD or emesis.

**Table 1:** Sociodemographic characteristics, comorbidities, and lifestyle habits of the studied sample (N = 204).

Characteristics	*N* (%) or Mean ± SD
**Body mass index** (kg/m²) (*5 m.d.*)	42.9 ± 6.6
**Females**	147 (72)
**Age** (years) (*5 m.d.*)	47.4 ± 13.7
**No salary or monthly allowance** (*6 m.d.*)	63 (31)
**Dyspnoea or sleep apnea**	173 (85)
**Diabetes status** (*1 m.d.*)	
None	159 (78)
Controlled	29 (14)
Uncontrolled	15 (7)
**GERD or emesis**	54 (27)
**Sweet or acidic diet** (*11 m.d.*)	80 (39)
**Smoking status** (*8 m.d.*)	
Never smokers	122 (60)
Past smokers	32 (16)
Current smokers	42 (21)
**Alcohol consumption** (*10 m.d.*)	13 (6)

GERD: gastroesophageal reflux disorder; m.d.: missing data; N: number; SD: standard deviation; Uncontrolled diabetes: glycated hemoglobin >7%.

As shown in Table 2, about 80% of participants reported having a regular dentist, with a mean time since the last dental visit of 19 ± 32 months. Dental pain at screening was reported by 18% of patients, and the screening revealed that 92% of participants needed dental treatment. With regard to hygiene practices, almost all participants owned a toothbrush and toothpaste, but more than one-third reported brushing their teeth <2 times/day. Use of other dental hygiene products was limited (30% used mouthwash; 13% interdental brushes; 11% floss; and <4% dental water jet). Debris was observed in 36% of participants, calculus in 76%, and 11% had mucosal lesions. On average, participants had at least one mobile tooth, ~33% had gingival recessions, and 36% had bleeding gums. The mean DMFT was 12 ± 7 including a mean number of 6 ± 5 filled teeth, 4 ± 6 missing teeth, and 1 ± 2 decayed teeth. About half of the sample (53%) had one or more untreated decayed teeth. Eight patients (4%) had a null DMFT (no caries experience). Among roots exposed in the oral cavity, more than 10% had a carious lesion. Missing teeth were generally not replaced, such that 13% of participants had masticatory inefficiencies. About 25% of participants were diagnosed with low tooth wear, and more severe wear was uncommon.

**Table 2:** Oral health status and related practices of the studied sample (N = 204).

Characteristics	*N* (%) or Mean ± SD
**Regular dentist**	163 (80)
**Time since last dental visit** (months)	18.6 ± 31.8
**Dental pain at screening** (*2 m.d.*)	37 (18)
**Need for oral treatment**	187 (92)
**Toothbrush owner**	201 (99)
**Toothpaste user**	199 (98)
**Brush less than twice a day**	71 (35)
**Mouthwash user**	62 (30)
**Interdental brushes user** (*1 m.d.*)	27 (13)
**Floss user**	23 (11)
**Dental water jet user** (*2 m.d.*)	8 (4)
**Presence of debris** (*1 m.d.*)	73 (36)
**Presence of calculus** (*2 m.d.*)	155 (76)
**Mucosal lesions**	22 (11)
**No. of mobile teeth** (*7 m.d.*)	1.3 ± 3
**Gingival recession**	67 (33)
**Gingival bleeding** (*2 m.d.*)	73 (36)
**Tooth decay ≥ 1**	108 (53)
**No. of decayed teeth**	1.3 ± 1.9
**No. of filled teeth**	6.2 ± 5.0
**No. of missing teeth**	4.1 ± 5.7
**No. of non-replaced missing teeth**	2.5 ± 4.0
**No. of missing teeth replaced by removable dentures**	1.3 ± 4.3
**No. of missing teeth replaced by fixed prosthodontics**	0.2 ± 0.7
**DMFT**	11.6 ± 7.1
**Root Caries Index**	11.4 ± 29.7
**Masticatory inefficiency** (*1 m.d.*)	26 (13)
**Tooth wear** (*2 m.d.*)	
None	149 (73)
Low	51 (25)
Medium	2 (1)

DMFT: number of decayed, missing, and filled teeth (0–28 points); m.d.: missing data; SD: standard deviation.

With regard to saliva parameters, resting flow rate was prolonged for 21% of participants, and about 40% had bubbly or sticky saliva. Insufficient stimulated flow rate was observed in about 33% of participants; 18% had acidic saliva, and more than half had low or very-low buffering capacity (Table 3).

**Table 3:** Saliva parameters of the studied sample (N = 204).

Characteristics	Overall (*n* = 204)	
**Resting flow rate >30 s** (*9 m.d.*)	42	(21)
**Consistency** (*9 m.d.*)		
Clear	117	(57)
Bubbly	61	(30)
Sticky	17	(8)
**Stimulated flow rate** (*9 m.d.*)		
>5 mL	129	(63)
3.5–5.0 mL	39	(19)
<3.5 mL	27	(13)
**pH** (*9 m.d.*)		
6.8–7.8	158	(78)
5.0–6.6	37	(18)
**Buffering capacity** (*9 m.d.*)		
Normal (10–15)	81	(40)
Low (6–9)	94	(46)
Very low (0–5)	20	(10)

Values in *N* (%). m.d.: missing data

**Figure 1:**
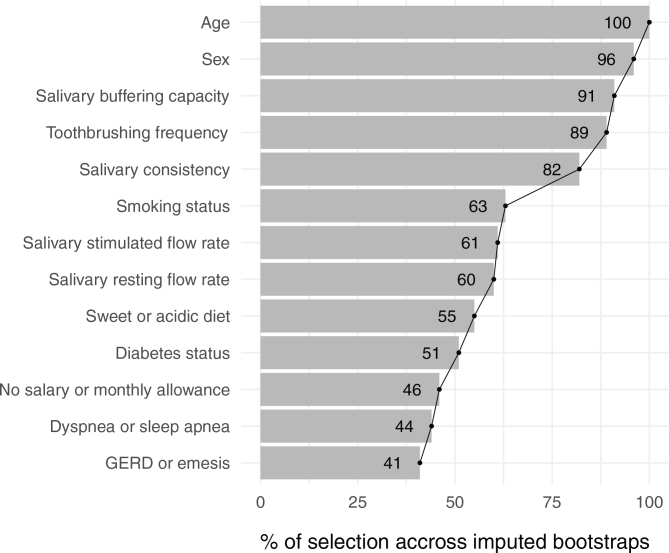
Characteristics of patients with obesity associated with the number of decayed, missing, and filled teeth (*N* = 204). Minimax concave penalty for linear regression. GERD: gastroesophageal reflux disorder.

Figure 1 shows the consistency of selection of patient characteristics for their association with the DMFT across imputed bootstraps in the MCP model. According to the elbow criterion, five variables selected more than 80% of the time were retained: age, sex, salivary buffering capacity, toothbrushing frequency, and salivary consistency. In post hoc unpenalised regression analysis, these variables were significantly associated with the DMFT (Table 4). On average, the DMFT increased with age (for one SD increase: β, 0.23; 95% CI: 0.16–0.29) and in females (β, 3.85; 95% CI, 1.77–5.93). Additionally, participant toothbrushing <2 times/day was associated with a mean increase in the DMFT of 2.87 (0.90–4.85) points compared with those who brushed ≥2 times/day. The average DMFT of participants with low and very-low salivary buffering capacity was 2.05 (0.14–3.96) and 3.78 (0.80–6.76) points higher than participants with physiological buffering capacity, respectively. Finally, participants with bubbly saliva had a mean increase in the DMFT of 2.45 (0.52–4.38) points compared with those who had clear saliva.

**Table 4:** Exploratory post hoc unpenalized linear regression with patient characteristics identified as most relevant to the number of decayed, missing, and filled teeth in the MCP model (N = 204).

Variables	Mean estimated difference in the number of decayed, missing, and filled teeth*		*P*
**Age** (for 1 SD increase)^†^	0.23	(0.16–0.29)	<0.001
**Sex** (female vs. male)	3.85	(1.77–5.93)	<0.001
**Salivary buffering capacity**			0.020
Normal (10–15)	ref		
Low (6–9)	2.05	(0.14–3.96)	
Very low (0–5)	3.78	(0.80–6.76)	
**Brush less than twice a day** (y vs. n)	2.87	(0.90–4.85)	0.005
**Salivary consistency**			0.038
Clear	ref		
Bubbly	2.45	(0.52–4.38)	
Sticky	0.20	(−3.00–3.39)	

* β (95% CI).

† 1 standard deviation (SD) = 13.69 years.

## Discussion

Our results revealed a high burden of pain, debris, calculus, periodontal problems, dental caries, and masticatory inefficiency in this sample of French patients with obesity attending a therapeutic education programme. A significant number of participants also reported poor oral health care in terms of the regularity of dental visits and individual oral hygiene. Furthermore, brushing frequency <2 times/day, older age, female sex, low buffering capacity, and a bubbly consistency of saliva were identified as the most relevant factors associated with the lifetime caries experience.

In line with the oral health resolution of the 2021 World Health Assembly, these results underscore the importance of maintaining oral health promotion efforts, even in high-income countries such as France [1]. Based on our results, this is especially important in patients with obesity. The mean DMFT and the prevalence of dental caries were high in our sample, and similar rates have been found in other high-income populations with obesity, including Australia [25] and South Korea [26]. Dental caries is largely preventable through simple and cost-effective measures, such as regular toothbrushing with fluoride [27]. Although access to preventive care products did not appear to be an issue in this study (almost all patients reported owning a toothbrush and toothpaste), patients tended not to engage in the recommended frequency of toothbrushing, which was a key factor associated with the caries experience in this study, as extensively described in the literature [28]. The prevalence of toothbrushing twice a day or more was indeed lower in this study than reported in the general French adult and adolescent populations (65% vs. 78%, respectively) [29, 30]. This result underlines the need for more appropriate preventive interventions to develop this habit.

The association between age and the caries experience was expected because dental caries is a chronic condition and the DMFT is a cumulative index representing the lifetime experience. The increasing burden of dental caries over time can, however, be mitigated with appropriate preventive practices.

With regard to the relationship between sex and the caries experience, the higher burden of dental caries in women has been previously described in the general adult population [31, 32]. Several hypotheses have been put forward to explain these differences: earlier tooth eruption, hormonal influences on saliva, or societal differences between genders regarding oral health [31, 32]. Previous research has also revealed that females may be more prone to snacking than males [33], especially in France [34]. Repeated food intake during the day can multiply exposure to carbohydrates or acidic foods, increasing the number of periods of demineralisation of the teeth and thus the risk of dental caries [7]. Unfortunately, snacking behaviour of the participants was beyond the scope of this study.

Finally, with regard to decreased buffering capacity of saliva, we observed an upward trend in the mean DMFT that was independent of age and other saliva parameters. Physiologically, bicarbonate, phosphate, and proteins in saliva exert a buffering action that neutralises dietary and biofilm acids, attenuating demineralisation of the tooth [35, 36]. An altered buffering capacity therefore facilitates the development of tooth decay. Notably, this factor affected more than half of the study sample. Chronic inflammation and oxidative stress observed in patients with obesity could indeed induce dysfunction of the salivary glands and change the composition of the saliva, in particular by modifying the protein and phosphate contents [15, 37–41]. Together, our findings suggest that these changes in composition may influence the consistency of saliva, and that this may be associated with dental caries. However, we found no previous study that addressed these issues. We also observed a high prevalence of a reduced salivary flow rate and acidic pH, as previously observed in populations with obesity [10–13, 39]. These changes could be due to specific obesity-related comorbidities or medications. Diabetes, for example, can alter the composition of saliva [42]. The literature indeed tends to show that the saliva of people with diabetes have a lower flow rate, buffering capacity, and calcium, phosphate, and fluoride contents [43–45]. In pre-clinical studies, different physio-pathological changes in animals, such as insulin resistance, obesity and diabetes, induced different changes in the lipid and protein profiles of the saliva [46, 47]. Changes in the metabolic profile of the saliva have also been identified in human with diabetes [48, 49]. We did not find a direct association between diabetes and the caries experience in our study, but we did observe associations with salivary parameters, which might suggest an indirect saliva-mediated relationship. Regarding the drugs, those for gastrointestinal disorders, cardiovascular diseases, psycholeptics, and anti-histamines, among others, which are drugs commonly used by people with obesity, have been reported to induce salivary gland hypofunction [50]. However, the impact of these drugs on salivary parameters other than the flow rate, such as its composition, is poorly described. The extent of the changes observed also seems to vary from study to study, suggesting that further research is needed to better understand the salivary gland dysfunctioning, and the impact of comorbidities and medications, in people with obesity.

This study had some limitations, including its cross-sectional design, which does not distinguish to what extent factors constitute a risk, a consequence, or a concomitant phenomenon of experiencing caries. The sample size, although reasonable, could be increased to improve estimation accuracy. Further studies with longitudinal designs and larger sample sizes are thus necessary to clarify these findings. Additionally, the sample population was hospitalised patients, which raises questions about its representativity of the general population. Regarding the oral examination, because data collection was carried out during a screening programme and not initially for research purpose, the intra-examiner agreement was not evaluated. Periodontitis was not evaluated as this would require a time-consuming examination of the periodontium. However, the presence of mobile teeth, gingival recessions, and gingival bleeding were used as proxies to assess periodontal health. Regarding the sweet/acidic diet and the absence of a salary or monthly allowance, non-quantitative definitions might explain why they were not selected in the MCP model as key factors related to the caries experience [51, 52]. Additionally, the food frequency questionnaire used in this study, and the categories adopted, were introduced to roughly estimate participants’ exposure to sugar and acidity, and therefore gives a rather approximate picture of their diet. Finally, the present analyses were exploratory rather than confirmatory, but they yielded interesting hypotheses on a set of characteristics associated with dental caries in people with obesity.

This study had several strengths as well. It is the first to evaluate the oral health status and related practices of adults with obesity in France, and the findings highlight the need for more preventive educational interventions in this high-income population. The administration of the questionnaire by an interviewer and use of statistical imputation limited the number of participants who were excluded from analyses due to missing data (only 12 of 216 participants who consented to the oral screening). Another strength is our innovative statistical approach: MCP enabled consideration of all potential factors related to dental caries in a single model to select the most relevant to this association. Numerous factors were therefore studied, including saliva parameters, while residual confounding factors may still exist.

The findings of this study serve as a reminder that serious gaps remain concerning the prevention of oral diseases, even in high-income countries. Indeed, a high caries burden was observed in this sample of French adults with obesity, a population at high risk for dental caries. The caries experience was significantly related to a brushing frequency of less than twice a day, as well as older age, female sex, low buffering capacity, and the bubbly consistency of the saliva. An oral health education prevention programme improving patient competence in their own oral care, such as twice-daily toothbrushing and targeting the youngest age groups, in particular females, could therefore limit the burden of dental caries with advancing age among people with obesity by acting against modifiable risk factors. Finally, our results indicate that saliva may be altered in people with obesity thus affecting teeth health; more research is needed to explore the underlying mechanisms.

## Supplementary Material

Association of socio-demographic characteristics, comorbidities, lifestyle habits, and saliva parameters with dental caries in adults with obesity
