# Supplementary material for: Association of socio-demographic characteristics, comorbidities, lifestyle habits, and saliva parameters with dental caries in adults with obesity
Source: Acta Odontol Scand. 2025 Jan 6;84:42485. doi: 10.2340/aos.v84.42485 (PMC11734531; doi:10.2340/aos.v84.42485)
Supplement: Association of socio-demographic characteristics, comorbidities, lifestyle habits, and saliva parameters with dental caries in adults with obesity [file AOS-84-42485-s1.pdf]

Supplementary material has been published as submitted. It has not been copyedited or typeset by Acta Odontologica Scandinavica.

Supplementary Information

Methods S1. Food frequency questionnaire completed by the participants. Bordeaux (France), 2014-2015.

How often do you eat the following foods?

	Never	Occasionally	Weekly (at least once a week)	Daily (at least once a day)
**Sugar, honey**				
**Sweets** (candies, chocolate bars, pastries)				
**Sweetened milks**				
**Fruit compotes**				
**Spreads, jams**				
**Ice cream**				
**Sweetened medicines**				
**Fast food**				
**Citrus fruits** (orange, lemon, grapefruit)**, rhubarb**				
**Dressing, mustard, pickle**				

How often do you drink the following beverages?

	Never	Occasionally	Weekly (at least once a week)	Daily (at least once a day)
**Water**				
**Milk**				
**Fruit juices** (lemon, orange, …)				
**Sodas** (Cola, Sprite, Fanta, …)				
**Energy drinks** (Gatorade, Powerade, Redbull, Monster, …)				
**Hot drinks** (coffee, tea, …)				
**Wine**				

Methods S2. Complete methodology for identifying the characteristics of patients with obesity related to sociodemographic profile, comorbidities, lifestyle habits or saliva parameters, most associated with the number of decayed, missing, and filled teeth (DMFT). Bordeaux (France), 2014-2015.

Step 1: Chi-square or Fisher's exact tests were used among explanatory variables to identify dependency. Alcohol consumption (strongly associated with smoking status) and salivary pH (strongly associated with other saliva parameters) were excluded.

Step 2: To check the stability of variable selection in step 4, 1,000 bootstrap samples were generated by random sampling with replacement from the original data set.

Step 3: missing data were imputed using multiple imputation by chained equations for the 22 patients with data missing completely at random (no report from the medical staff in the medical records or loss of collected data). These missing data occurred for age (3% of participants), salary or monthly allowance (3%), diet (5%), smoking status (4%), diabetes (1%), and salivary parameters (4%). Imputation was thus performed 10 times with 10 iterations cycles on each of the 1,000 bootstrap samples with the *mice* R package, resulting in 10,000 bootstrap imputed datasets.

Step 4: The distribution of the DMFT appears Gaussian (Figure S1). Therefore, using the *grpreg* R package, a minimax concave penalty (MCP) for linear regression was repeated on each of the 10,000 bootstrap imputed datasets to identify patient characteristics most associated with the caries experience in this sample. MCP is indeed an alternative to LASSO (Least Absolute Shrinkage and Selection Operator) which are both penalized methods that shrink the regression coefficients towards zero without compromising the residual sum of square, resulting in variable selection; but MCP compensates for the drawbacks of LASSO (i.e. consistency in simultaneous selection and asymptotic unbiasedness (23)). Quantitative exposures were standardized before implemented MCP to avoid some variables to be more penalized than others due to their magnitude. The tuning parameter (which influence the strength of the shrinkage) was chosen for each model as the value that minimizes the cross-validation error after performing a 10-fold cross-validation. Moreover, each model across the 10,000 imputed bootstraps was performed with a different seed. Bootstrap resampling (step 2) and change of seed enable to check the stability of variable selection and thus improve precision in the

identification of patient characteristics most related to carious experience. Indeed, percentages of selection were calculated across imputed bootstraps.

Step 5: using the elbow criterion, we retained variables that preceded a sharp decline on the graphical representation of the percentages of selection across imputed bootstraps. Next, to improve our understanding of these findings, exploratory *post hoc* unpenalized linear regression with these variables as explanatory factors were performed on the imputed original dataset. Residuals were checked graphically to test the normality and homoscedasticity assumptions.

Figure S1. Distribution of the number of decayed, missing, and filled teeth (DMFT) in the studied sample (N=204). Bordeaux (France), 2014-2015.

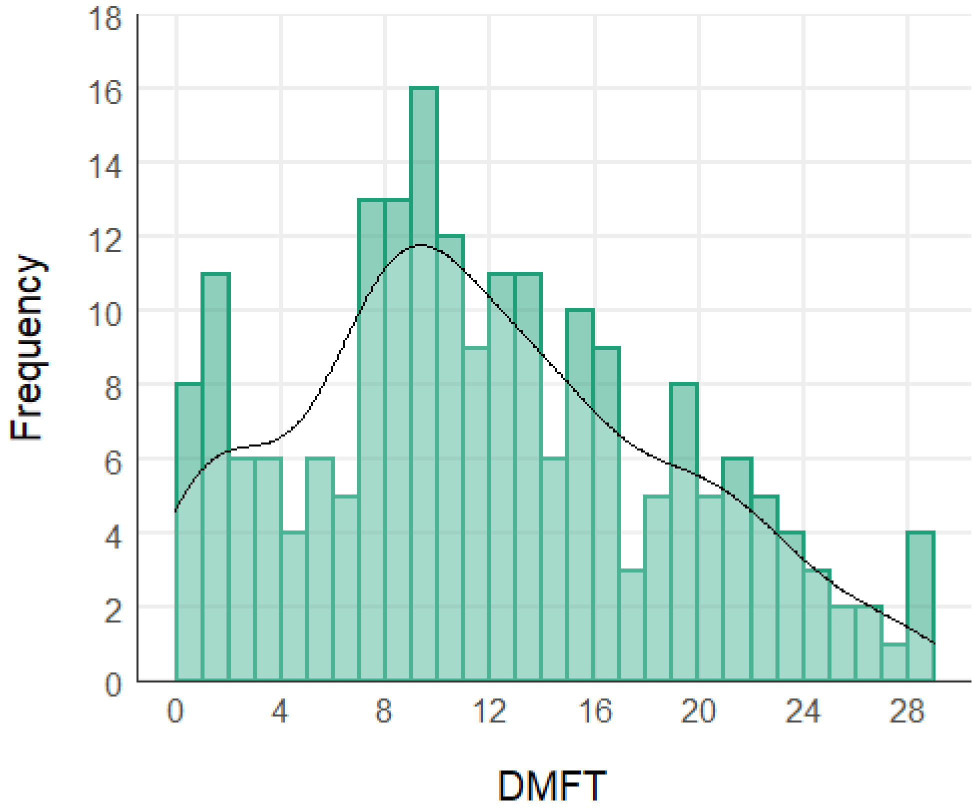

Table S1. Sociodemographic characteristics of the participants included in the study compared with those not included. Bordeaux (France), 2014-2015.

	Included sample N=204	Not included sample N=112	
Characteristics	N (%) or Mean ± SD	N (%) or Mean ± SD	*P*
**Body mass index** (kg/m²)	42.9 ± 6.6	43.7 ± 7.2	0.340
**Females**	147 (72)	85 (76)	0.461
**Age** (years)	47.4 ± 13.7	50.0 ± 12.2	0.097
**No salary or monthly allowance**	63 (31)	34 (30)	0.952
**Dyspnea or sleep apnea**	173 (85)	10 (9)	<0.001
**Diabetes status** (*1 m.d.*)			<0.001
None	159 (78)	110 (98)	
Controlled	29 (14)	1 (1)	
Uncontrolled	15 (7)	1 (1)	
**GERD or emesis**	54 (27)	2 (2)	<0.001

N: number; SD: standard deviation.
